# Dietary Histidine, Threonine, or Taurine Supplementation Affects Gilthead Seabream (*Sparus aurata*) Immune Status

**DOI:** 10.3390/ani11051193

**Published:** 2021-04-21

**Authors:** Lourenço Ramos-Pinto, Marina Machado, Josep Calduch-Giner, Jaume Pérez-Sánchez, Jorge Dias, Luís E. C. Conceição, Tomé S. Silva, Benjamín Costas

**Affiliations:** 1 Centro Interdisciplinar de Investigação Marinha e Ambiental (CIIMAR), Universidade do Porto, Terminal de Cruzeiros do Porto de Leixões, Avenida General Norton de Matos, S/N, 4450-208 Matosinhos, Portugal; mcasimiro@ciimar.up.pt; 2 Instituto de Ciências Biomédicas Abel Salazar (ICBAS-UP), Universidade do Porto, Rua de Jorge Viterbo Ferreira no. 228, 4050-313 Porto, Portugal; 3 SPAROS Lda., Área Empresarial de Marim, Lote C, 8700-221 Olhão, Portugal; jorgedias@sparos.pt (J.D.); luisconceicao@sparos.pt (L.E.C.C.); TomeSilva@sparos.pt (T.S.S.); 4 Nutrigenomics and Fish Growth Endocrinology, Institute of Aquaculture Torre de la Sal, IATS-CSIC, 12595 Castellón, Spain; j.calduch@csic.es (J.C.-G.); jaime.perez.sanchez@csic.es (J.P.-S.)

**Keywords:** amino acids, immune-modulation, mucosal immunity, blood leucocytes

## Abstract

**Simple Summary:**

The concept of supporting animal health through the best possible nutrition is well-accepted in modern aquaculture, and functional amino acids (AAs) appear to be good candidates to improve health and growth performance. For instance, histidine (His), taurine (Tau), and threonine (Thr) appear to play important roles in homeostatic maintenance, detoxification of reactive species, and immune function. The present study aimed to evaluate the effects of His, Tau, and Thr supplementation on the gilthead seabream (*Sparus aurata*) immune status. In general, the results suggest that dietary supplementation with His, Tau, or Thr above their nominal requirements for this species has relatively mild effects. Still, some effects of threonine and taurine supplementation on the fish immune system were observed, particularly after a short-term feeding period (four weeks), which reinforces the importance of feeding period when aiming to improve immune alertness. Hence, further studies with other supplementation levels and eventually duration of supplementation could help to clarify the potential immunomodulatory role of these AAs for gilthead seabream.

**Abstract:**

AAs have become interesting feed ingredients to be used in functional fish feeds as not only are they protein building blocks, but they also participate in several other key metabolic processes. In the present study, a comprehensive analysis of transcriptomics, hematology, and humoral immune parameters (plasma and skin mucus) were measured twice over the course of the feeding trial (four weeks). Plasma antiprotease activity increased in fish fed Thr compared to those fed the CTRL and Tau treatments, regardless of sampling time. The bactericidal activity in skin mucus decreased in fish fed Tau and His treatments compared to those fed the CTRL diet after two weeks. The membrane IgT (*mIgT*) was upregulated in fish fed Tau after four weeks, while C-type lectin domain family domain 10 member (*clec10a*) was downregulated in fish fed Thr after two weeks of feeding. By comparing the molecular signatures of head-kidney by means of a PLS-DA, it is possible to visualize that the main difference is between the two sampling points, regardless of diet. Altogether, these results suggest that dietary supplementation with these AAs at the tested levels causes mild immune-modulation effects in gilthead seabream, which should be further studied under disease challenge conditions.

## 1. Introduction

In a fish farming context, fish are susceptible to a wide range of pathogens, since seawater is a good growing media for many bacteria and virus that can ruin an entire fish stock. Once present in water, pathogens are in direct contact with the first lines of defense of fish and can easily spread, particularly if the animals are not well-nourished and prepared [1,2]. As such, health maintenance is of utmost importance in modern fish farming, and establishing strategies to improve fish immune status and welfare is essential.

Recently, there has been an effort to adopt the use of supplements that can boost fish immune status (e.g., yeast extracts, probiotics, prebiotics, and amino acids) in the development of functional aquafeeds, which can contribute to significantly reduce the abusive use of antimicrobials (e.g., antibiotics) and disinfectants [3]. Functional diets are those that extend beyond satisfying the basic nutritional requirements of the farmed fish, contributing toward optimal growth, health, and survival through the inclusion of specific additives with health and growth-promoting properties [4]. Moreover, it is known that amino acid (AA) requirements are expected to increase under challenging rearing conditions, so dietary AA supplementation beyond the nominal requirements for each species could benefit animals (e.g., improved immune status and increased tolerance to environmental stresses), as already reviewed elsewhere [5,6]. Thus, research on dietary AA supplementation as potential functional additives (i.e., supplied at levels beyond the species’ requirements) in fish, with particular emphasis on their effects on the immune system, should be better explored.

Histidine (His) is an essential AA (EAA) abundant in plasma albumin and skeletal muscle in fish [7]. His participates in one-carbon unit metabolism, consequently affecting DNA and protein synthesis [8]. Directly or through its derivative compounds, histidine plays an important role in homeostatic maintenance, osmoregulation, muscle pH buffering, and detoxification of reactive carbonyl species [9,10]. In humans, His also contributes significantly to the buffering capacity of plasma and tissue proteins [11]. The protein buffer effect is the result of their dissociable side groups. For most proteins including hemoglobin, the most important of these dissociable groups is the imidazole ring of His residues. The His metabolite carnosine (beta-alanyl-L-histidine) also combats intramuscular acidosis by maintaining intracellular and extracellular buffering in muscle tissue pH [12]. Moreover, various studies indicate that His and its derivatives act as antioxidants or can mitigate the impact of oxidative stress [13,14,15]. Histidine and its imidazole derivatives such as carnosine and anserine have been proven to scavenge reactive oxygen species, and also contribute to improve taste, texture, and overall fillet quality [9,11,16]. Additionally, a study with juvenile grass carp fed a His-deficient diet demonstrated significantly increased osmotic fragility of erythrocytes [16]. Thus, histidine supplementation is expected to modulate both fish antioxidant status and immune response.

Threonine (Thr) is often considered the third limiting AA after lysine and methionine for growing fish fed low fishmeal feed formulations [17,18]. It is also involved in many physiological and biochemical processes including growth and immune functions [18,19,20]. Duval, et al. [21] discovered that Thr-enriched cell culture medium prevented apoptosis, stimulated mouse hybridoma cell growth, and promoted antibody production in lymphocytes through protein synthesis and cellular signaling mechanisms. Additionally, Thr deficiency upregulated nitric oxide levels in blood monocytes of broilers [22]. Dietary Thr inclusion at 13.9 mg/g diet improved growth, digestive and absorptive capacity as well as the antioxidant status in intestine and hepatopancreas of sub-adult grass carp [23]. Thr is also a major component of mucin in the small intestine of animals, which suggests its importance in the regulation of intestinal barrier integrity and function [24,25]. Therefore, the expectation is that a dietary Thr surplus will modulate fish mucosal health.

Taurine (Tau) is a conditionally essential nutrient that, although technically considered as an amino sulfonic acid due to its chemical structure [7,26], is often referenced to as an amino acid (in the sense of a small aminated acid). Tau can be synthesized from methionine and cysteine in fish, but the rate of synthesis is usually too low to satisfy the nutritional requirements of (at least) carnivorous fish [27]. Hence, growth depression is one of the first clear observed signs reported during Tau deficiency [26]. In fact, Tau supplementation in rainbow trout (*Oncorhynchus mykiss*) fed a fishmeal-free diet improved growth and feed efficiency [28]. Taurine’s role across numerous biological processes in fish has already been described, with a range of physiological problems and histological changes having been reported when Tau levels are reduced in the diet, namely green liver syndrome, reduced hematocrit, anemia, and reduced disease resistance [26]. Taurine has also been described to play an important osmoregulatory role in fish [29]. At the moment, information suggesting that Tau plays a role in fish health and immunity is still very fragmented. Nevertheless, Maita, et al. [30] reported that yellowtail (*Seriola quinqueradiata*) fed a fishmeal-free diet supplemented with Tau displayed improved fish survival in response to an artificial bacterial challenge, reaching similar levels as those fed a fishmeal-containing control diet, suggesting it may have immunoregulatory properties in fish. Hence, taurine supplementation is expected to possibly improve fish growth and feed efficiency, while modulating the immune system.

Several seabream species are farmed worldwide due to their savory meat and to meet its growing consumption trend. Among Sparidae, the gilthead seabream (*Sparus aurata*, L.) is one of the main carnivorous farmed fish species in the Mediterranean region [31]. A sustainable and profitable aquaculture relies on the production of healthy fish, which requires balanced feeds manufactured with high-quality ingredients [32,33]. The main goal of this study was to evaluate the effects of His, Thr, and Tau on gilthead seabream (*Sparus aurata*) immune function when added as a supplement to a practical aquafeed formulation during a transient (two weeks) and short-term (four weeks) feeding period.

## 2. Materials and Methods

### 2.1. Diet Formulations

Extruded feeds were based on plant proteins sources and limiting fish meal inclusion to 12%. This mimics most of the currently used commercial diets for gilthead seabream. Using this basal formulation, three other experimental diets were produced at SPAROS Lda. (Olhão, Portugal) through the inclusion of crystalline His, Thr, and Tau.

Briefly, a control (CTRL) diet was formulated to meet current commercial formulations for this species as well as its known nutritional requirements. Three other diets were identical to the CTRL diet but supplemented with 0.4% His, 0.75% Thr, or 0.5% of Tau of feed (Table 1). These inclusion levels were chosen to be at least 50% above the requirement levels established for gilthead seabream [34,35]. A single common base formulation was prepared and extruded, with the four different diets being obtained by the application of different vacuum coatings post-extrusion: the CTRL diet was subjected to vacuum coating with oil, while the other three (TAU, HIS, and THR) were coated with a mixture of oil and the corresponding AA, in order to attain the target AA supplementation levels. Main ingredients were ground (below 250 μm) in a Hosakawa, model #1 micropulverizer hammer mill (Hosokawa Micron Ltd., Preston Brook-United Kingdom). These ground ingredients were then mixed according to the target formulation in a Double-helix Mixture TGC, model 500 L (TGC Extrusion, Roullet-Saint-Estèphe, France), to attain a basal mixture (no oils were added at this stage). All diets were manufactured by extrusion (pellet size 2.0 mm) by means of a pilot-scale twin-screw extruder CLEXTRAL BC45 (Clextral, Firminy, France) with a screw diameter of 55.5 mm and temperature ranging 105–110 °C. Upon extrusion, all batches of extruded feeds were dried in a convection oven (OP 750-EF, LTE Scientifics, Greenfield, UK) for 2 h at 60 °C. After this process, pellets were left to cool at room temperature, and subsequently the AA (taurine, histidine, and threonine for each of the correspondent experimental diets) were mixed with the fish oil fraction according to each target formulation and added under vacuum coating conditions in a Pegasus vacuum mixer (PG-10VCLAB, Dinnisen, Sevenum, Limburg, The Netherland). Proximate composition analysis was performed by the following methods: dry matter, by drying at 105 °C for 24 h; ash, by combustion at 550 °C for 12 h; crude protein (N × 6.25), by a flash combustion technique followed by gas chromatographic separation and thermal conductivity detection (LECO FP428); fat, after petroleum ether extraction, by the Soxhlet method; total phosphorus, according to the ISO/DIS 6491 method, using the vanado-molybdate reagent; and gross energy, in an adiabatic bomb calorimeter (IKA).

Total AA content of diets was determined by hydrolyzation in 6 M HCl at 116 °C for 2 h in nitrogen-flushed glass vials. Samples were then pre-column derivatized with Waters AccQ Fluor Reagent (6-aminoqui-nolyl-N-hydroxysuccinimidyl carbamate) using the AccQ Tag method (Waters, Milford, MA, USA). Analyses were done by ultra-high performance liquid chromatography in a Waters reverse-phase AA analysis system using norvaline as an internal standard. During acid hydrolysis, asparagine is converted to aspartate and glutamine to glutamate, so the reported values for these AAs represent the sum of the respective amine and acid. The resultant peaks were analyzed with EMPOWER software (Waters, Milford, MA, USA). Tryptophan was independently determined by HPLC, after alkaline hydrolysis (Silliker Portugal, S.A. Vila Nova de Gaia-Portugal) The AA profiles of the experimental diets are presented in Table 2.

### 2.2. Rearing Conditions

The current trial was conducted by trained scientists (following FELASA category C recommendations) and according to the animal experimentation guidelines on the protection of animals used for scientific purposes from the European directive 2010/63/UE at the experimental facilities of I3S (Instituto de Investigação e Inovação em Saúde, Porto, Portugal).

Gilthead seabream juveniles with an average weight of 8.77 ± 0.44 g were acquired from a certified hatchery (SONRÍONANSA; Cantabria, Spain) and were randomly distributed into 12 tanks (200 L; *n* = 70) for an acclimatization period of three weeks being fed the CTRL diet. Fish were held in a recirculation seawater system in which oxygen concentration (7.3 ± 0.01 mg L^−1^) and photoperiod were automatically controlled (10 h dark and 14 h light). Temperature was maintained by a water heater/cooler system at 20 ± 0.5 °C and kept unchanged throughout the experiment. Both nitrite and ammonium levels were daily recorded, and its levels controlled by a water ozonizer system. Water renovations and system cleanings were performed twice a week. After the quarantine period, the experiment was started by feeding each group (three tanks) with the respective feed three times a day *ad libitum*. The control (CTRL) group was fed a control diet, which met the EAA requirement levels estimated for gilthead seabream [34,35]. The other three groups were fed diets with the inclusion of Thr, Tau, or His (THR, TAU, and HIS dietary treatments, respectively), as stated in Table 2 for one month. No deaths during the experimental trial were observed.

### 2.3. Experimental Procedures

An initial sampling point was set, and 10 fish fed the CTRL diet were euthanized and designated as time zero (TØ) to assess their immune status prior to the feeding trial.

The feeding trial lasted four weeks in order to assess the effect of short- and mid-term dietary AA supplementation. Feed intake was recorded daily and body weight of fish fed dietary treatments was measured at the beginning and at the end of the feeding trial. Growth was monitored by obtaining the initial body weight (IBW) and final body weight (FBW) and used to calculate growth performance parameters.

Zootechnical performance measures were calculated as:

Relative growth rate (RGR, %.day^−1^ = (e^g^ − 1) × 100

where g = (ln (Final body weight) − ln (Initial body weight))/days, calculated as a tank average.

At two and four weeks after feeding the experimental diets, 36 fish from each group (i.e., 12 per replicate) were euthanized by anesthetic overdose with 2-phenoxyethanol and individually weighed. Samples were obtained for immunological and transcriptomic studies. Skin mucus was collected by gently scraping the fish dorsal-lateral surface using a cell scraper with enough care to avoid contamination with blood, urogenital, and intestinal excretions, snap frozen in liquid nitrogen and stored at −80 °C. Blood was collected from the caudal vein using heparinized syringes, centrifuged at 10,000× *g* for 10 min and plasma pools were stored. Blood from four fish per tank was also used to perform blood smears. Head-kidney was collected and snap frozen for gene expression analysis. All samples were immediately kept at −80 °C until further processing.

### 2.4. Hematological Procedures

Blood was collected from the caudal vein using heparinized syringes. The hematological profile consisted of total white (WBC) and red (RBC) blood cells counts and blood smears for differential WBC counting as described by Machado, et al. [36].

Blood smears were initially fixed with formol-ethanol (formaldehyde at 4%) and afterward stained with Wright’s stain (Haemacolor; Merck, Darmstadt, Germany). Neutrophils were identified according to their peroxidase activity, which was detected using the method described by Afonso, et al. [37]. The slides were examined under oil immersion (1000×) and at least 200 leucocytes per slide were counted and classified as thrombocytes, lymphocytes, monocytes, and neutrophils. The relative proportion of each cell type was subsequently calculated.

### 2.5. Immune Parameters

Plasma bactericidal activity was measured using *Vibrio anguillarum* according to Graham, et al. [38], adapted by Machado, Azeredo, Diaz-Rosales, Afonso, Peres, Oliva-Teles and Costas [36], with some modifications. Succinctly, 20 µL of plasma was added as duplicate to wells of a U-shaped 96-well plate. Hanks’ Balanced Salt solution (HBSS) was added to four wells instead of plasma and served as a positive control. To each well, 20 µL of *V. anguillarum* (1 × 10^6^ cfu mL^−1^) were added and the plate was incubated for 3 h at 25 °C. To each well, 25 µL of iodonitrotetrazolium chloride, INT (2-(4-iodophenyl)-3-(4-nitrophenyl)-5-phenyl-2H-tetrazolium chloride; 1 mg.mL^−1^; Sigma-Alrich, Steinheim am Albuch, Germany) was added to allow the formation of formazan. Plates were then centrifuged at 2000× *g* for 10 min and the precipitate was dissolved in 200 µL of dimethyl sulfoxide (Sigma). The absorbance of the dissolved formazan was measured at 490 nm in a Synergy HT microplate reader (Biotek, Winooski, Vermont, USA). Total bactericidal activity is expressed as the percentage of killed bacteria, calculated from the difference between the samples (surviving bacteria) and the positive control (100% living bacteria).

IgM in plasma was measured by an Enzyme-Linked Immunosorbent Assay (ELISA assay). Succinctly, 4 µL of plasma were previously diluted (1:100) in 396 µL of Na_2_CO_3_ (50 mM, pH = 9.6) buffer and then 100 µL of these diluted plasma samples were added to the 96 well in duplicates using 100 µL of buffer (Na_2_CO_3_) as a negative control (added to four wells). The samples (antigen) were allowed to stand at 22 °C for 1 h, being subsequently removed by means of an aspirator with a pipet tip. Then, 300 µL of blocking buffer (5% low fat milk powder in 0.1% Tween 20) was added to each well and incubated for 1 h at 22 °C. This mixture was then removed by aspiration and followed by three consecutive washes with 300 µL of T-TBS (0.1% Tween 20). T-TBS (0.1% Tween) is a solution made with 20 mM Tris Base (Nzytech) and 137 mM NaCl (Sigma), distilled water, and TWEEN 20 (Sigma). After properly cleaning and drying the wells, 100 µL of the anti-gilthead seabream primary IgM monoclonal antibody previously diluted in blocking buffer (1:100) was added to each well and incubated for 1 h at 22 °C. The primary antibody was then removed by aspiration, with three consecutive washes being performed. Afterward, the anti-mouse IgG-HRP secondary antibody diluted 1:1000 in blocking buffer was added and incubated for 1 h at 22 °C, then being recovered by aspiration. The wells were then washed three times and 100 µL of 3,3′,5,5′-tetramethylbenzidine hydrochloride (TMB) substrate solution for ELISA (BioLegend #421101), previously prepared, was added to each well and incubated for 5 min. The color change reaction was stopped after 5 min by adding 100 µL of 2 M sulfuric acid and the optical density was read at 450 nm.

The antiprotease activity was determined as described by Ellis [39] adapted by Machado, Azeredo, Diaz-Rosales, Afonso, Peres, Oliva-Teles and Costas [36]. Briefly, 10 µL of plasma were incubated with the same volume of trypsin solution (5 mg.mL^−1^ in NaHCO_3_, 5 mg.mL^−1^, pH 8.3) for 10 min at 22 °C in polystyrene microtubes. To the incubation mixture, 100 µL of phosphate buffer (NaH_2_PO_4_, 13.9 mg.mL^−1^, pH 7.0) and 125 µL of azocasein (20 mg.mL^−1^ in NaHCO_3_, 5 mg.mL^−1^, pH 8.3) were added and incubated for 1 h at 22 °C. Finally, 250 µL of trichloroacetic acid were added to each microtube and incubated for 30 min at 22 °C. The mixture was centrifuged at 10,000× *g* for 5 min at room temperature. Afterward, 100 µL of the supernatant was transferred in duplicates to a 96-well plate that previously contained 100 µL of NaOH (40 mg.mL^−1^) per well. The OD was read at 450 nm. Phosphate buffer was added to some wells instead of plasma and trypsin and served as a blank, whereas the reference sample was phosphate buffer instead of plasma. The percentage of inhibition of trypsin activity compared to the reference sample was calculated.

Total peroxidase activity in plasma was measured according to the procedures described by Quade and Roth [40]. Briefly, 15 µL of plasma in duplicates were diluted with 135 µL of HBSS without Ca^2+^ and Mg^2+^ in flat-bottomed 96-well plates. Then, 50 µL of 20 mM 3,3′,5,5′-tetramethylbenzidine hydrochloride (TMB; Sigma, Alrich, Steinheim am Albuch, Germany) and 50 mL of 5 mM H_2_O_2_ were added. The color change reaction was stopped after 2 min by adding 50 mL of 2 M sulfuric acid and the optical density was read at 450 nm. Wells without plasma were used as blanks. One unit of peroxidase activity (units.mL^−1^ plasma) was defined by the quantity of peroxidase that produces an absorbance change of 1 OD.

### 2.6. Gene Expression Analysis

Head-kidney were taken from fish fed the experimental diets for two and four weeks. Genes were analyzed using the seabream PCR-array platform of the IATS Nutrigenomics group (http://nutrigroup-iats.org accessed on 7 March 2021). Total RNA was extracted using a MagMAX^TM^-96 total RNA Isolation Kit (Life Technologies, Carlsbad, CA, USA). RNA yield was 50–100 μg with 260 to 280 nm UV absorbance ratios (A260/280) of 1.9–2.1. Reverse transcription (RT) of 500 ng total RNA was performed with random decamers using a High-Capacity cDNA Reverse Transcription Kit (Applied Biosystems, Foster City, CA, USA), according to the manufacturer’s instructions. Negative control reactions were run without reverse transcriptase.

Real-time quantitative PCR was carried out on a CFX96 Connect^TM^ Real-Time PCR Detection System (Bio-Rad, Hercules, CA, USA) using a 96-well PCR array layout designed for simultaneously profiling of a panel of 29 genes for head-kidney (summarized by biological processes in Table 3). Selected genes were associated with health biological processes, like interleukins and cytokines (9), macrophage and monocyte chemokines (3), immunoglobulins (4), antiprotease (1), antimicrobial peptide/iron recycling (1), T-cell markers (6), and pattern recognition receptors (5). Controls of general PCR performance were included on each array with all the pipetting operations performed by means of the EpMotion 5070 Liquid Handling Robot (Eppendorf, Hamburg, Germany). Briefly, RT reactions were diluted to convenient concentrations and the equivalent of 660 pg of total input RNA was used in a 25 μL volume for each PCR reaction.

PCR-wells contained a 2× SYBR Green Master Mix (Bio-Rad) and specific primers at a final concentration of 0.9 μM were used to obtain amplicons of 50–150 bp in length (Table S1). The program used for PCR amplification included an initial denaturation step at 95 °C for 3 min, followed by 40 cycles of denaturation for 15 s at 95 °C and annealing/extension for 60 s at 60 °C. The efficiency of PCR reactions was always higher than 90%, and negative controls without sample templates were routinely performed for each primer set. The specificity of reactions was verified by analysis of melting curves (ramping rates of 0.05 °C/s over a temperature range of 55–95 °C), and linearity of serial dilutions of RT reactions. Fluorescence data acquired during the PCR extension phase were normalized using the delta–delta Ct method [41]. β-Actin was tested for gene expression stability using GeNorm software (M score = 0.21) and used as a housekeeping gene in the normalization procedure. Fold-change calculations were done in reference to the mean response of CTRL fish. To compare the mRNA gene expression level of a panel of genes in a given dietary treatment, all data values were expressed in reference to the expression level of *casp3* in CTRL fish, which was arbitrarily assigned a value of 1.

### 2.7. Statistical Analysis

All results were expressed as means ± standard error (SE). Univariate statistic evaluation of the data was accomplished by two-way ANOVA with sampling time and dietary treatment as main factors. A significance of *p* < 0.05 was applied to all statistical tests. Gross deviations from the ANOVA assumptions of error normality and homoscedasticity were evaluated through residual analysis (using QQ-plots and “residuals vs. fitted” scatter plots). All tests were run with SPSS statistical analysis software (SPSS ver.26.0; Chicago, IL, USA).

For gene expression data, unsupervised principle component analysis (PCA) was first performed on data as an unbiased statistical method to observe intrinsic trends in the dataset using EZ-INFO^®^ v3.0 (Umetrics, Malmö, Sweden). To achieve the maximum separation between groups, supervised partial least-squares discriminant analysis (PLS-DA) was subsequentially applied. Potential differential genes were selected according to the Variable Importance in the Projection (VIP) values. Variables with VIP > 1 were considered to be influential for the separation of samples in PLS-DA analysis [42,43,44].

## 3. Results

### 3.1. Growth Performance

No differences among dietary groups were observed either for final body weight (FBW) or relative growth rate (RGR) at the end of the feeding trial (Table 4).

### 3.2. Immune Parameters

Total red blood cells (RBC) and white blood cells (WBC) counts increased between the first and second sampling point, regardless of dietary treatments. Lower WBC counts were observed for fish fed THR after two weeks compared to their counterparts fed the other dietary treatments (Table 5). Similarly, peripheral lymphocytes were less abundant in fish fed HIS than those fed THR after four weeks, while circulating neutrophils increased in seabream fed TAU compared to fish fed His, regardless of sampling time.

### 3.3. Plasma Humoral Parameters

Most plasma humoral immune parameters changed between sampling points, regardless of dietary treatments. Bactericidal and antiprotease activities increased after four weeks, while IgM levels had the opposite pattern. Regarding dietary effects, plasma antiprotease activity augmented in fish fed THR compared to seabream fed CTRL and TAU dietary treatments, regardless of sampling time (Table 6).

### 3.4. Skin Mucus Humoral Parameters

The bactericidal activity in skin mucus decreased in seabream fed TAU and HIS dietary treatments compared to their counterparts fed the CTRL diet after two weeks of feeding, displaying levels of mucosal bactericidal activity similar to ones measured in TØ fish. Mucus antiprotease activity increased in seabream fed HIS compared to fish fed the CTRL diet regardless of sampling time (Table 6).

### 3.5. Gene Expression

**Figure 1:**
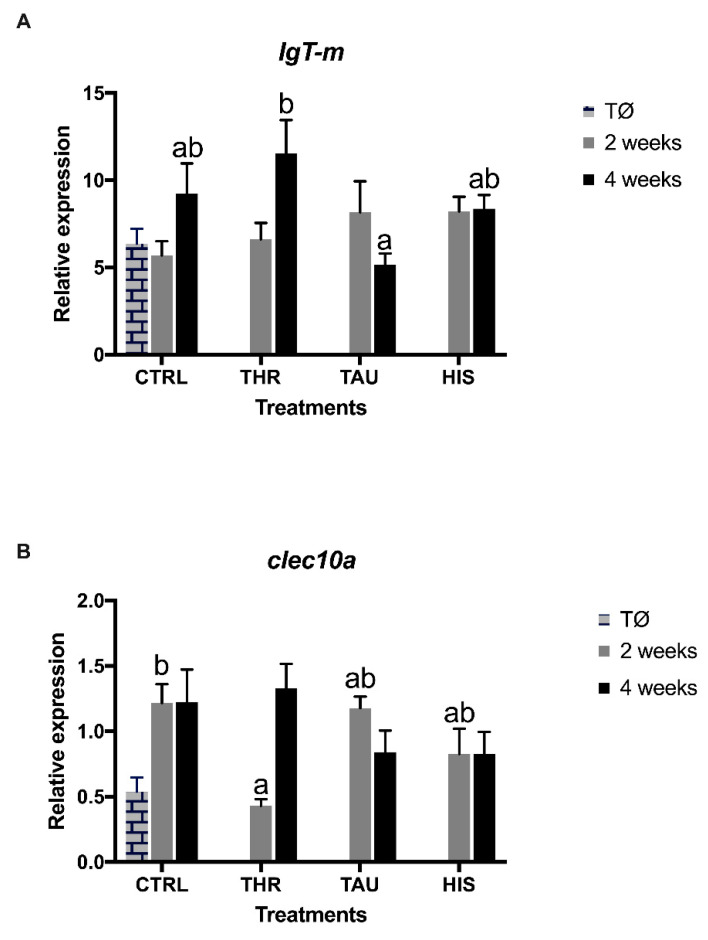
Relative expression of Immunoglobulin T membrane-bound form (*IgT-m*, (**A**) C-type lectin domain family 10 member A (*clec10a*, (**B**) genes in the head-kidney of gilthead seabream at time Ø and fed the dietary treatments during two and four weeks. Values are presented as means ± SE (*n* = 9). *p*-values from two-way ANOVA (*p* ≤ 0.05). Tukey’s post-hoc test was used to identify differences in the experimental treatments. a,b: Different lowercase letters stand for significant differences among dietary treatments for the same sampling time. treatment.control (CTRL), histidine (His), taurine (Tau), and threonine (Thr)

Gene expression seems to be consistent with the humoral parameters measured in plasma and skin mucus. *IgT-m* decreased significantly in seabream fed TAU compared to those fed THR dietary treatments for a 4-week feeding period (Figure 1A and Table S2). The C-type lectin domain family domain 10 member (*clec10a*) decreased in seabream fed THR dietary treatment compared to fish with the CTRL diet after two weeks of feeding (Figure 1B and Table S2).

Through the PLS-DA analysis considering all nine groups resulting from the combinations of all experimental factors (Figure S1A) and the one with only four groups (diet effect) (Figure S1B), a low degree of explained variance was observed, coupled with a low prediction capacity: with nine groups, we obtained R^2^Y of 11% and Q^2^ of 0%, while a reduction to four increased the R^2^Y to 14% and the Q^2^ remained 0%. In fact, by reducing the number of groups to two (2W and 4W), the discriminant analysis (Figure S1C) was able to explain 54% of variance (R^2^ Y) and predict more than 32% of the total variance (Q^2^). In order to understand and interpret the contribution of the different genes to these components, a table of the variable importance projection (VIP) score of the genes is presented in Figure S1D, ordered by their importance. The VIP values indicated the relevant contribution (VIP > 1) of 11 genes for this group differentiation. It was also clear that most genes (8/11) contributed to component 1 (which explained 33.11% of total variance) differentiation, along which the most differentiation between time groups was observed.

## 4. Discussion

The present study showed that some modulation of leucocyte response was observed in fish fed a TAU dietary treatment, with the number of circulating neutrophils being increased regardless of sampling time. There is no published information about Tau involvement in differentiation of this cell-type, but it is known that neutrophils contain very high concentrations of taurine due to its uptake from the blood [45]. Hence, some stimulation of Tau levels on neutrophil numbers could be postulated and it could also be speculated that Tau requirement may increase upon infection. Neutrophils from the reserve pools are quickly attracted to infectious foci by microbial products and chemotactic substances released by host cells [46].

Moreover, plasma antiprotease increased in fish fed THR dietary treatment compared to those fed the CTRL diet, which could indicate a potential capacity of this particular AA to boost the host’s innate immune defenses. Antiproteases play a crucial role in the inhibition of certain proteases either by “trapping” proteases to avoid protein hydrolysis or by binding to their active sites [47,48]. The observed effect of a dietary Thr surplus may also translate into an improved resistance against bacteria in animals fed higher levels of this AA. Additionally, several pathogens (mainly bacteria) express a variety of non-specific proteases that degrade important proteins involved in fish innate immunity [49,50]. Hence, the presence of stronger antiprotease activity in seabream fed the THR dietary treatment may indicate an improvement in fish immune condition.

Skin mucus is an important physical and chemical barrier against invading pathogens. It serves as an important component of the innate immune machinery by: (i) its continuous renewal, preventing stable colonization of the skin by pathogens; and (ii) containing a number of innate immune proteins and enzymes (e.g., lysozyme, complement proteins, and antibacterial proteins and petites) including antiproteases [51]. In the present study, seabream fed HIS dietary treatment showed an increase of total antiprotease activity measured in skin mucus. In contrast, fish fed the HIS diet had a lower bactericidal activity in skin mucus compared to those fed the CTRL diet after two weeks, which seems to point to a differential immune status in fish fed this diet. One should take into account that the bactericidal activity is a multifactorial indicator since it evaluates a wide range of innate immune mechanisms and molecular defenses against bacterial invasion such as proteins of the complement system, antimicrobial peptides, acute phase proteins, immunoglobulins, lysozyme, and cytokines [38,52]. Therefore, and considering the decrease in lymphocyte numbers observed at four weeks, we suggest that dietary His supplementation can improve certain key aspects of innate immunity in detriment of factors contributing to acquired immunity at the skin mucus level.

The results obtained in terms of bactericidal activity are comparable with those of previous works where the impact of a Tau-rich diet on lysozyme and alternative complement system was evaluated. Han, et al. [53] observed that Tau supplementation did not influence blood parameters (e.g., hematocrit, hemoglobin, glucose, total protein, total cholesterol, total bilirubin, and triglycerides) of Japanese flounder (*Paralichthys olivaceus*). This was corroborated by Magalhães, et al. [54] in a similar study with white seabream (*Diplodus sargus*) juveniles, where no differences were observed in the plasma alternative complement pathway and lysozyme levels. Nonetheless, a diet rich in taurine boosted the lysozyme activity and other immune parameters such as phagocytic index, respiratory burst, and total immunoglobulin content, in yellow catfish (*Pelteobagrus fulvidraco*) [55]. This highlights the importance of further studies to clarify the effects of Tau on fish immune system, since it could be species and context-dependent.

In the present study, dietary treatments did not induce strong transcriptional changes, as the observed differences were mostly time-dependent. Nonetheless, some mild effects were observed with the downregulation of *clec10,* a member of the C-type lectin (CTLs) superfamily, in seabream fed the THR dietary treatment for two weeks.

Lectins are a family of glycoprotein pattern-recognition receptors highly represented in fish, typically being multivalent proteins that recognize and bind specific carbohydrate recognition domains (CRD) [56]. The presence of several CRDs, in combination with other proteins domains, enables not only the recognition of specific carbohydrates on the pathogen’s surface, but also on the surface of immunocompetent cells. Some lectins induce the synthesis of pro-inflammatory cytokines including *il1-β1*, *il1-β2*, *tnf-α1*, *tnf-α2*, and *il8* in rainbow trout macrophages and fibroblast-like cells [57]. Lectins play an active role in innate immunity in PAMP recognition, opsonization, phagocytosis, and complement activation [58,59]. C-type lectins, in particular, have several functions in innate immunity and contribute to the homing of leucocytes and immune cell trafficking as well as pathogen recognition and subsequent T cell activation [60]. As such, CLEC10 is likely to play a role in regulating adaptive and innate immune responses and, thus, in this study, the downregulation of this CTL in seabream fed THR diet for two weeks could be suggested, and the concomitant decrease in blood total WBC, as a possible inhibition of immune alertness (e.g., pathogen recognition and innate immunity).

Overall, some unexpected variation in the AA concentrations measured in feeds were observed. On the other hand, given that all diets were produced from the same basal formulation, we expected true differences in non-supplemented AA to be smaller than that measured. Thus, we expected the observed effects to be mostly attributable to the increase in threonine, histidine, and taurine.

Moreover, Ramos-Pinto, Azeredo, Silva, Conceição, Dias, Montero, Torrecillas, Silva and Costas [32] described that arginine and citrulline, particularly when supplemented at a 1% of feed inclusion level, induced a stimulation of the fish immune system after a short-term feeding period, verified mostly by a modulation of the gilthead seabream plasma proteome and health-biomarkers after four weeks of exposure. Hence, the fact that arginine concentration was higher in the CTRL diet compared to the others could have translated into an increased gilthead seabream immune status. It can therefore be speculated that an eventual immune modulation in fish fed the CTRL diet due to higher arginine levels could have attenuated more positive effects regarding dietary threonine, histidine, or taurine supplementation.

In the present study, data from the analyzed responses of fish fed supplemented diets suggest that the effects of Thr, His, and Tau supplementation on fish immune system seem to be of an indirect nature, when compared with other EAAs previously studied such as tryptophan, methionine, or arginine [32,61,62], which present stronger and more direct effects. In a previous work where the effects of dietary tryptophan supplementation were also explored in gilthead seabream, only a transient immune enhancement was observed in fish fed an extreme formulation (no FM inclusion) over a short-term feeding period (two weeks), suggesting that these putative advantageous effects seem to disappear over a longer feeding period (13 weeks) [33]. The present study also underlines the fact that “feeding time”, besides “dosage”, seems to be a crucial factor in determining the AA efficacy. It seems that supplements, in this case AA, may lose their effectiveness after being available for a long feeding period. A possible explanation can be assumed: fish becomes adapted to this supplementation level through some physiological/metabolic readjustment. Therefore, the importance of “exposure time” as a central factor in determining the apparent functional effects of additives seems to be of major importance. In the present study, this fact seems to be particularly relevant for the THR dietary treatment. While a decrease in total WBC numbers and *clec10* transcripts was observed after two weeks of feeding, the acquired arm of the immune system was stimulated by dietary Thr supplementation with an enhancement of peripheral lymphocytes and *IgT-m* mRNA expression level in the head-kidney after four weeks of feeding.

## 5. Conclusions

This study suggests that dietary supplementation with His, Tau, or Thr at the tested levels causes mild immune-modulation effects in gilthead seabream, which should be further studied under disease challenge conditions. Still, plasma antiprotease activity increased in fish fed THR dietary treatment which, together with a decrease in *clec10* transcripts and the total WBC at two weeks, in contrast with an enhancement of acquired immune condition at four weeks. This reinforces the importance of feeding time when aiming to improve immune alertness. In addition, HIS dietary treatment led to an increase in total antiproteases activity measured in skin mucus, while some stimulation of dietary Tau on peripheral neutrophil numbers could also be seen. Hence, further studies optimizing His, Tau, and Thr supplementation regarding dose (different supplementation levels), feeding periods and timings, under challenging scenarios (e.g., bacterial challenge or stress), could help to clarify the potential immunomodulatory role of these AAs for gilthead seabream.

## Figures and Tables

**Table 1:** Ingredients of the experimental diets.

Ingredients (% Feed Basis)	Experimental Diets			
	CTRL	HIS	TAU	THR
Fishmeal LT70 (NORVIK) ^a^	12.00	12.00	12.00	12.00
Poultry meal 65 ^b^	5.00	5.00	5.00	5.00
Soy protein concentrate (Soycomil) ^c^	8.50	8.50	8.50	8.50
Wheat gluten ^d^	7.37	7.37	7.37	7.37
Corn gluten ^e^	8.00	8.00	8.00	8.00
Soybean meal 48 ^f^	7.68	7.68	7.68	7.68
Soybean meal 44 ^g^	15.00	15.00	15.00	15.00
Rapeseed meal ^h^	5.00	5.00	5.00	5.00
Wheat meal ^i^	14.30	14.30	14.30	14.30
Sardine oil ^j^	4.65	4.65	4.65	4.65
Rapeseed oil ^k^	10.85	10.85	10.85	10.85
Vit & Min Premix INVIVO 1% ^l^	1.00	1.00	1.00	1.00
Antioxidant ^m^	0.20	0.20	0.20	0.20
Sodium propionate ^n^	0.10	0.10	0.10	0.10
MCP ^o^	0.20	0.20	0.20	0.20
DL-Methionine ^p^	0.15	0.15	0.15	0.15
L-Threonine ^q^				0.75
L-Histidine ^r^		0.40		
Taurine ^s^			0.50	
**Proximate analyses**				
Dry matter (% feed)	94.6	94.3	94.2	94.3
Crude protein (% dry weight)	42.62	41.92	42.24	42.23
Crude lipid (% dry weight)	18.3	19.1	18	18.3
Ash (% dry weight)	6.4	6.1	6.3	6.1
Gross Energy (kJ g^−1^ DM)	21.83	21.75	21.37	22.00

^a^ Fish meal LT70: 71.9%CP (crude protein), 6.8% CF (crude fat), Norvik Sopropêche, France ^b^ Poultry meal: 65%CP, 14.4% CF, SAVINOR UTS, Portugal ^c^ Soycomil P: 63% CP, 0.8% CF, ADM, The Netherlands ^d^ Wheat gluten: 80.4% CP; 5.6% CF, VITAL Roquette, France ^e^ Corn gluten meal: 61% CP, 6% CF, COPAM, Portugal. ^f^ Soybean meal 48: Solvent extracted dehulled soybean meal: 47% CP, 2.6% CF, CARGILL, Spain ^g^ Soybean meal 44: Solvent extracted dehulled soybean meal: 44% CP, 1.8% CF, CARGILL, Spain ^h^ Rapeseed meal: Defatted rapeseed meal: 37.7% CP, 2.3% CF, Premix Lda, Portugal ^i^ Wheat meal: 11.7% CP; 1.6% CF, Casa Lanchinha, Portugal ^j^ Sardine oil, 98.1% CF, Norvik Sopropêche, France ^k^ Rapeseed oil, 98.2% CF Henry Lamotte Oils GmbH, Germany ^l^ Vitamin and mineral premix: INVIVONSA Portugal SA, Portugal: Vitamins (IU or mg/kg diet): DL-alpha tocopherol acetate, 100 mg; sodium menadione bisulfate, 25 mg; retinyl acetate, 20,000 IU; DL-cholecalciferol, 2000 IU; thiamin, 30 mg; riboflavin, 30 mg; pyridoxine, 20 mg; cyanocobalamin, 0.1 mg; nicotinic acid, 200 mg; folic acid, 15 mg; ascorbic acid, 500 mg; inositol, 500 mg; biotin, 3 mg; calcium panthotenate, 100 mg; choline chloride, 1000 mg, betaine, 500 mg. Minerals (g or mg/kg diet): copper sulphate, 9 mg; ferric sulphate, 6 mg; potassium iodide, 0.5 mg; manganese oxide, 9.6 mg; sodium selenite, 0.01 mg; zinc sulphate, 7.5 mg; sodium chloride, 400 mg; excipient wheat gluten. ^m^ Antioxidant: VERDILOX, Kemin Europe NV, Belgium ^n^ Sodium propionate: Disproquímica, Portugal ^o^ Monocalcium phosphate: Premix Lda, Portugal ^p^ DL-Methionine: DL-METHIONINE FOR AQUACULTURE 99%, EVONIK Nutrition & Care GmbH, Germany ^q^ L-Threonine: ThreAMINO 98.5%, Evonik Nutrition & Care GmbH, Germany ^r^ L-Hisdidine: L-Histidine 98%, Ajinomoto Eurolysine SAS, France ^s^ L-Taurine: L-Taurine 98%, ORFFA, The Netherlands. control (CTRL), histidine (His), taurine (Tau), and threonine (Thr).

**Table 2:** Amino acid (AA) composition (g AA 100 g^−1^ CP) of the experimental diets.

AA	Experimental Diets			
	CTRL	HIS	TAU	THR
Arginine	7.6	6.1	6.3	5.7
Histidine	2.1	3.4	2.6	2.4
Lysine	5.3	6.3	6.7	6.2
Threonine	3.8	3.5	3.6	4.3
Isoleucine	4.5	4.2	4.3	4.1
Leucine	8.4	7.2	7.1	6.9
Valine	4.8	4.9	5.0	4.7
Methionine	2.3	2.5	2.9	2.2
Phenylalanine	5.3	4.0	4.0	3.8
Cystine	0.6	1.3	1.3	1.3
Tyrosine	4.0	3.4	3.5	3.1
Aspartic acid + Asparagine	7.4	9.6	10.1	9.5
Glutamic acid + Glutamine	19.0	21.8	19.0	21.9
Alanine	4.8	4.8	5.0	4.6
Glycine	5.1	4.4	4.9	4.2
Proline	6.7	6.6	6.5	6.4
Serine	4.5	4.4	4.5	4.4
Taurine	0.3	0.3	1.0	0.2

Tryptophan was not analyzed. control (CTRL), histidine (His), taurine (Tau), and threonine (Thr).

**Table 3:** Genes included in head kidney pathway-focused PCR array.

Gene Name/Category	Symbol	Gene Name/Category	Symbol
***Interleukins and cytokines***		***T-cell markers***	
Interleukin-1 beta	*il-1β*	Cluster of differentiation 3 zeta chain	*cd3x*
Interleukin-6	*il-6*	CD4-full	*cd4-full*
Interleukin-7	*il-7*	Cluster of differentiation 8 alpha	*cd8a*
Interleukin-8	*il-8*	Cluster of differentiation 8 beta	*cd8b*
Interleukin-10	*il-10*	Zeta-chain-associated protein kinase 70	*zap70*
Interleukin 12 subunit beta	*il12*		
Interleukin-15	*il-15*		
Interleukin-34	*il-34*	***Pattern recognition receptors***	
Tumor necrosis factor-alpha	*tnf α*	Toll-like receptor 2	*tlr2*
		Toll-like receptor 5	*tlr5*
***Macrophages and monocytes chemokines***		Toll-like receptor 9	*tlr9*
Macrophage colony-stimulating factor 1 receptor 1	*csf1r1*	Macrophage mannose receptor 1	*mrc1*
C-C chemokine receptor type 3	*ccr3*		
C-C chemokine CK8/C-C motif chemokine 20	*ck8/ccl20*	***Antimicrobial peptide/Iron recycling***	
		Hepcidin	*hepc*
***Immunoglobulins***			
Immunoglobulin M	*sIgM*	***Complement pathways***	
Immunoglobulin M membrane-bound form	*IgM-m*	Complement factor 3	*c3*
Immunoglobulin T	*sIgT*		
Immunoglobulin T membrane-bound form	*IgT-m*	***C-type lectin receptor signaling***	
		C-type lectin domain family 10 member A	*clec10a*
***Apoptosis***			
Caspase 3	*casp3*		

Colors in background is to distinguish the different pathways.

**Table 4:** Body weight (BW, g fish^−1^) and relative growth rate (RGR, % day^−1^) of gilthead seabream fed the experimental diets for two and four weeks.

**Parameters**	**CTRL**		**THR**		**TAU**		**HIS**			
	**2 Weeks**	**4 Weeks**	**2 Weeks**	**4 Weeks**	**2 Weeks**	**4 Weeks**	**2 Weeks**		**4 Weeks**	
FBW (g fish^-1^)	11.07 ± 0.23	14.49 ± 2.10	10.89 ± 0.30	14.04 ± 1.49	11.08 ± 0.33	13.89 ± 1.08	11.13 ± 0.49		13.70 ± 0.10	
RGR (RGR, % day^-1^)	1.63 ± 0.28	1.76 ± 0.50	1.65 ± 0.51	1.73 ± 0.35	1.54 ± 0.18	1.58 ± 0.32	1.84 ± 0.48		1.67 ± 0.09	
**Two-way ANOVA**										
**Parameters**	**Diet**	**Time**	**Time × diet**	**Time**		**Diet**				
				**2 weeks**	**4 weeks**	**CTRL**	**THR**	**TAU**		**HIS**
BW	0.925	<0.001	0.893	A	B	-	-	-		-
RGR	0.814	0.911	0.898	-	-	-	-	-		-

Values are presented as means ± SD (*n* = 12 and *n* = 3 for feed intake). *p*-values from two-way ANOVA (*p* ≤ 0.05). Tukey’s post-hoc test was used to identify differences in the experimental treatments. A,B: Different capital letters indicate differences between diets regardless of time or difference between times regardless of diets. Initial body weight was 8.77 ± 0.13 g.control (CTRL), histidine (His), taurine (Tau), and threonine (Thr), final body weight (FBW).

**Table 5:** Hemoglobin, red blood cells (RBC), and white blood cells (WBC) of gilthead seabream fed dietary treatments during two and four weeks.

**Parameters**		**CTRL**	**CTRL**		**THR**		**TAU**		**HIS**			
		**TØ**	**2 Weeks**	**4 Weeks**	**2 Weeks**	**4 Weeks**	**2 Weeks**	**4 Weeks**	**2 Weeks**		**4 Weeks**	
Hemoglobin	(g dL^−1^)	0.60 ± 0.10	0.60 ± 0.06	0.60 ± 0.05	0.65 ± 0.09	0.65 ± 0.04	0.57 ± 0.03	0.56 ± 0.05	0.54 ± 0.06		0.67 ± 0.10	
WBC	(×10^4^ µL)	4.66 ± 0.33 ^b^	4.53 ± 0.60 ^b^	6.21 ± 0.36	3.34 ± 0.20 ^a^	7.11 ± 0.56	4.85 ± 0.28 ^b^	7.00 ± 0.40	4.94 ± 0.47 ^b^		5.94 ± 0.56	
RBC	(×10^6^ µL)	1.55 ± 0.15	1.14 ± 0.09	1.44 ± 0.10	1.28 ± 0.11	1.58 ± 0.04	1.00 ± 0.06	1.42 ± 0.09	1.24 ± 0.11		1.66 ± 0.12	
Absolute peripheral blood leucocytes												
Thrombocytes	(×10^4^ µL)	3.31 ± 0.23	3.46 ± 0.57	4.54 ± 0.26	2.46 ± 0.18	4.77 ± 0.36	3.62 ± 0.27	5.19 ± 0.40	3.56 ± 0.35		4.60 ± 0.47	
Lymphocytes	(×10^4^ µL)	1.21 ± 0.23 ^ab^	0.93 ± 0.14	1.37 ± 0.18 ^ab^	0.70 ± 0.34 *	1.95 ± 0.49 ^b#^	0.98 ± 0.46	1.49 ± 0.48 ^ab^	1.22 ± 0.49		1.11 ± 0.43 ^a^	
Monocytes	(×10^4^ µL)	0.02 ± 0.01	0.02 ± 0.01	0.06 ± 0.02	0.02 ± 0.01	0.06 ± 0.02	0.04 ± 0.01	0.08 ± 0.02	0.07 ± 0.02		0.06 ± 0.02	
Neutrophils	(×10^4^ µL)	0.13 ± 0.03	0.07 ± 0.02	0.22 ± 0.04	0.08 ± 0.03	0.18 ± 0.02	0.18 ± 0.03	0.25 ± 0.02	0.09 ± 0.01		0.15 ± 0.04	
**Two-way ANOVA**												
**Parameters**		**Diet**	**Time**	**Time × diet**	**Time**			**Diet**				
					**TØ**	**2 weeks**	**4 weeks**	**CTRL**	**THR**	**TAU**		**HIS**
Hemoglobin	(g dl ^−1^)	0.633	0.538	0.699	-	-	-	-	-	-		-
WBC	(×10^4^ µL)	0.406	<0.001	0.017	A	A	B	-	-	-		-
RBC	(×10^6^ µL)	0.286	<0.001	0.898	B	A	B	-	-	-		-
*Absolute peripheral blood leucocytes*												
Thrombocytes	(×10^4^ µL)	0.214	<0.001	0.298	A	A	B	-	-	-		-
Lymphocytes	(×10^4^ µL)	0.74	<0.001	0.003	AB	A	B	-	-	-		-
Monocytes	(×10^4^ µL)	0.071	0.003	0.116	AB	A	B	-	-	-		-
Neutrophils	(×10^4^ µL)	0.006	<0.001	0.287	A	A	B	AB	AB	B		A

Values are presented as means ± SE (*n* = 9). *p*-values from two-way ANOVA (*p* ≤ 0.05). Tukey’s post-hoc test was used to identify differences in the experimental treatments. a, b: Different lowercase letters stand for significant differences between dietary treatments for the same time. A, B: Different capital letters indicate differences between diets regardless of time or difference between times regardless of diets. Different symbols indicate difference between times for the same dietary treatment. control (CTRL), histidine (His), taurine (Tau), and threonine (Thr).

**Table 6:** Plasma and mucus humoral parameters of gilthead seabream fed dietary treatments during two and four weeks.

**Parameters**		**CTRL**	**CTRL**		**THR**		**TAU**			**HIS**			
		**TØ**	**2 Weeks**	**4 Weeks**	**2 Weeks**	**4 Weeks**	**2 Weeks**	**4 Weeks **		**2 Weeks**			**4 Weeks**
Plasma													
Bactericidal activity	(%)	28.57 ± 7.31	43.75 ± 2.14	47.56 ± 3.01	37.65 ± 2.56	48.25 ± 2.76	38.47 ± 2.20	39.92 ± 3.30		42.39 ± 3.40			43.54 ± 2.90
Antiprotease	(%)	**⨂**	92.02 ± 0.99	92.69 ± 1.43	94.60 ± 0.59	95.45 ± 1.01	90.06 ± 0.85	94.56 ± 0.59		91.71 ± 1.04			95.97 ± 0.50
Peroxidase	(U/mL)	**⨂**	6.29 ± 0.71	7.07 ± 0.71	6.60 ± 0.79	7.18 ± 0.55	6.34 ± 0.64	6.44 ± 0.43		6.95 ± 0.43			7.94 ± 1.00
IgM	(abs)	0.80 ± 0.11	0.85 ± 0.02	0.72 ± 0.05	0.77 ± 0.04	0.68 ± 0.06	0.66 ± 0.04	0.74 ± 0.04		0.81 ± 0.05			0.66 ± 0.05
Mucus													
Bactericidal activity	(%)	60.53 ± 1.02 ^a^	66.41 ± 0.63 *^b^	55.83 ± 1.76 ^#^	64.57 ± 0.89 ^ab^	59.11 ± 1.59	60.80 ± 1.34 ^a^	59.08 ± 1.19		59.40 ± 1.26 ^a^			57.69 ± 1.49
Antiprotease	(%)	29.45 ± 2.37	25.52 ± 2.70	26.99 ± 1.33	28.10 ± 1.97	26.88 ± 1.31	28.36 ± 2.10	28.69 ± 2.49		27.52 ± 0.90			30.62 ± 2.6
Peroxidase activity	(U/mg)	0.66 ± 0.06	0.73 ± 0.09	0.69 ± 0.09	0.89 ± 0.10	0.78 ± 0.11	1.00 ± 0.12	0.71 ± 0.08		0.81 ± 0.11			0.78 ± 0.41
**Two-way ANOVA**													
**Parameters**		**Diet**	**Time**	**Time × diet**	**Time**			**Diet**					
					**TØ**	**2 weeks**	**4 weeks**	**CTRL**	**THR**		**TAU**	**HIS**	
Plasma													
Bactericidal activity	(%)	0.151	0.037	0.343	A	AB	B	-	-		-	-	
Antiprotease	(%)	0.013	<0.001	0.059	-	A	B	A	B		A	AB	
Peroxidase	(U/mL)	0.471	0.208	0.926	-	-	-	-	-		-	-	
IgM	(abs)	0.302	0.023	0.045	B	B	A	-	-		-	-	
Mucus													
Bactericidal activity	(%)	0.053	<0.001	0.001	AB	B	A	-	-		-	-	
Antiprotease	(%)	0.012	0.149	0.102	-	-	-	A	AB		AB	B	
Peroxidase activity	(U/mg)	0.452	0.1	0.541	-	-	-	-	-		-	-	

Values are presented as means ± SE (*n* = 12). *p*-values from two-way ANOVA (*p* ≤ 0.05). Tukey’s post-hoc test was used to identify differences in the experimental treatments. a, b: Different lowercase letters stand for significant differences between dietary treatments for the same time. A, B: Different capital letters indicate differences between diets regardless of time or difference between times regardless of diets. Different symbols indicate difference between times for the same dietary treatment. **⨂** There was not enough sample to perform this analysis due to fish size constraints. control (CTRL), histidine (His), taurine (Tau), and threonine (Thr).

## Data Availability

The data presented in this study are available on request from the corresponding author.

## Supplementary Materials

The following are available online at https://www.mdpi.com/article/10.3390/ani11051193/s1, Figure S1: Discriminant analysis (PLS-DA) of head-kidney molecular signatures of fishes fed the experimental diets. Relative expression data of the 29 genes included in the array can be found in Table 2. (A) PLS-DA scores plot of all biomarkers using “experimental group” as target factor for the two first components. (B) PLS-DA scores plot of all biomarkers using “diet” as target factor for the two first components. (C) PLS-DA scores plot of all biomarkers using “time” as target factor for the two first components (D) Ordered list of markers by variable importance (VIP) in projection of PLS-DA model for time differentiation. Markers with VIP values > 1 after the first and second components are highlighted in blue and yellow, respectively, Table S1: Forward (F) and reverse (R) primers used for real-time PCR in head kidney, Table S2: Head kidney expression in response in gilthead seabream at time Ø and fed dietary treatments for two weeks and four weeks. All values are reported as mean ± SE (*n =* 9) (raw data). P-values from two-way ANOVA (*p* ≤ 0.05). Tukey’s post-hoc test was used to identify differences in the experimental treatments. Different lowercase letters stand for significant differences between dietary treatments for the same time.
