# Supplementary material for: Dietary Histidine, Threonine, or Taurine Supplementation Affects Gilthead Seabream (Sparus aurata) Immune Status
Source: Animals (Basel). 2021 Apr 21;11(5):1193. doi: 10.3390/ani11051193 (PMC8143364; doi:10.3390/ani11051193)
Supplement: Supplementary file 1 [file animals-11-01193-s001.zip › animals-1156579-supplementary.pdf]

Supplementary Files

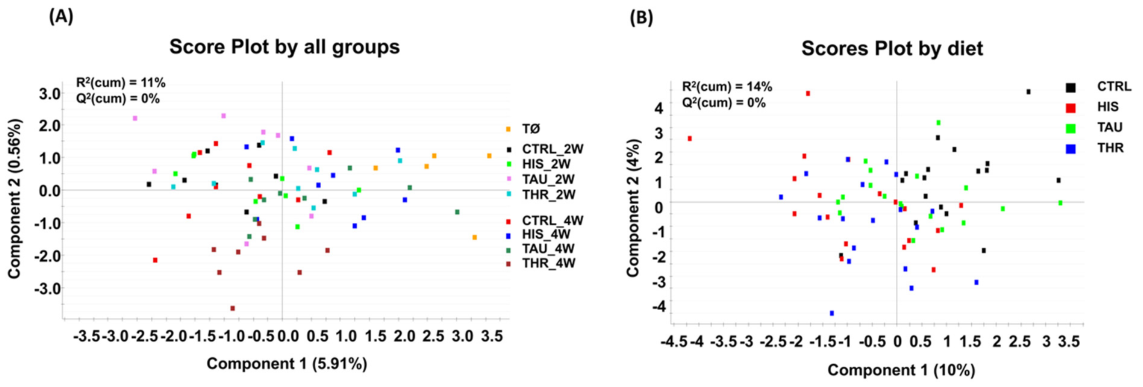

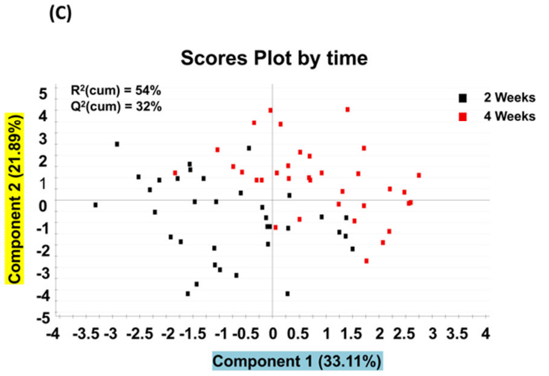

(D)

Order	Gene	VIP > 1 (after component 2)
1	*mIgT*	1.656
2	*ccr3*	1.591
3	*csf1r*	1.360
4	*casp3*	1.350
5	*mIgM*	1.327
6	*cd3x*	1.263
7	*mrc1*	1.211
8	*ck8/ccl20*	1.189
9	*il-34*	1.124
10	*c3*	1.066
11	*hepc*	1.064

Figure S1. Discriminant analysis (PLS-DA) of head-kidney molecular signatures of fishes fed the experimental diets. Relative expression data of the 29 genes included in the array can be found on table 2. (**A)** PLS-DA scores plot of all biomarkers, using "experimental group" as target factor, for the two first components. **(B)** PLS-DA scores plot of all biomarkers, using "diet" as target factor, for the two first components. **(C)** PLS-DA scores plot of all biomarkers, using "time" as target factor, for the two first components **(D)** Ordered list of markers by variable importance (VIP) in projection of PLS-DA model for time differentiation. Markers with VIP values > 1 after the first and second components are highlighted in blue and yellow, respectively.

Table S1. Forward (F) and reverse (R) primers used for real-time PCR in head kidney.

Gene Name	Symbol	Acc. No.		Primer sequences (5' → 3')
ß-actin	*actb*	X89920	F	TCCTGCGGAATCCATGAGA
			R	GACGTCGCACTTCATGATGCT
C-C chemokineCK8 / C-C motifchemokine 20	*ck8/ccl20*	GU181393	F	CCGTCCTCATCTGCTTCATACT
			R	GCTCTGCCGTTGATGGAAC
Caspase	*casp3*	EU722334	F	GCCAACGGACCTGGACCTG
			R	CCATCGCCTCTCCTCGCATCTA
C-C chemokine receptor type 3	*ccr3*	KF857317	F	CTACATCAGCATCACCATACGCATCCT
			R	TGGCACGGCACTTCTCCTTCA
CD4-full	*cd4-full*	AM489485	F	TCCTCCTCCTCGTCCTCGTT
			R	GGTGTCTCATCTTCCGCTGTCT
Cluster of differentiation 3 zeta chain	*cd3x*	MF175235	F	ATGGCGGTCCAGACGAGGGTTTC
			R	ACCAGCGAGGACAGGACCAGCAG
Cluster of differentiation 8 alpha	*cd8a*	EU921630	F	GCAGCAACGGTAACACGAACG
			R	CCAGTATGAGCGGAGTACAGAACA
Cluster of differentiation 8 beta	*cd8b*	KX231275	F	CCGAAATGTGGAAGACTGGAACTC
			R	CCAGTATGAGCGGAGTACAGAACA
Complement factor 3	*c3*	HM543456	F	GCTTACGCTCTTCTTGCTCTGGTGAA
			R	CATCTGACAACTGGTCTGGCATCGT
C-type lectin domain family 10 member A	*clec10a*	KF857329	F	CGACTCTGGACTCCCTCA
			R	CGTTGTTGATGGTGCGTTC
Hepcidin	*hepc*	AM749960	F	ACTCCTGGAAGATGCCGTATGC
			R	AACTTACACCTCCTGCGTCCAC
Immunoglobulin M	*igm*	JQ811851	F	ACCTCAGCGTCCTTCAGTGTTTATGATGCC
			R	CAGCGTCGTCGTCAACAAGCCAAGC
Immunoglobulin M membrane-bound form	*migm*	KX599199	F	GCTATGGAGGCGGAGGAAGATAACA
			R	CAGCGTCGTCGTCAACAAGCCAAGC
Immunoglobulin T	*igt*	KX599200	F	GCTGTCAAGGTGGCCCCAAAAG
			R	CAACATTCATGCGAGTTACCCTTGGC
Immunoglobulin T membrane-bound form	*igt-m*	KX599201	F	AGACGATGCCAGTGAAGAGGATGAGT
			R	CGAAGGAGGAGGCTGTGGACCA

Interleukin-1 beta	*il-1β*	AJ419178	F	GCGACCTACCTGCCACCTACACC
			R	TCGTCCACCGCCTCCAGATGC
Interleukin-6	*il-6*	EU244588	F	TCTTGAAGGTGGTGCTGGAAGTG
			R	AAGGACAATCTGCTGGAAGTGAGG
Interleukin-7	*il-7*	JX976618	F	CTATCTCTGTCCCTGTCCTGTGA
			R	TGCGGATGGTTGCCTTGTAAT
Interleukin-8	*il-8*	JX976619	F	CAGCAGAGTCTTCATCGTCACTATTG
			R	AGGCTCGCTTCACTGATGG
Interleukin-10	*il-10*	JX976621	F	AACATCCTGGGCTTCTATCTG
			R	GTGTCCTCCGTCTCATCTG
Interleukin 12 subunit beta	*il12*	JX976624	F	ATTCCCTGTGTGGTGGCTGCT
			R	GCTGGCATCCTGGCACTGAAT
Interleukin-15	*il-15*	JX976625	F	GAGACCAGCGAGCGAAAGGCATCC
			R	GCCAGAACAGGTTACAGGTTGACAGGAA
Interleukin-34	*il-34*	JX976629	F	TCTGTCTGCCTGCTGGTAG
			R	ATGCTGGCTGGTGTCTGG
Macrophage colony-stimulating factor 1 receptor 1	*csf1r1*	AM050293	F	TTGCGTGTGGTGAGGAAGGAAGGT
			R	AGCAGGCAGGGCAGCAGGTA
Macrophage mannose receptor 1	*mrc1*	KF857326	F	CTTCCGACCGTACCTGTACCTACTCA
			R	CGATTCCAGCCTTCCGCACACTTA
Toll-like receptor 2	*tlr2*	KF857323	F	CATCTGCGACTCTCCTCTCTTCCT
			R	GCGTGGATAGAGTTGGACTTGAG
Toll-like receptor 5	*tlr5*	KF857324	F	TCGCCAATCTGACGGACCTGAG
			R	CAGAACGCCGATGTGGTTGTAAGAC
Toll-like receptor 9	*tlr9*	AY751797	F	GCCTTCCTTGTCTGCTCTTTCT
			R	GCCGTAGAGGTGCTTCAGTAG
Tumor necrosis factor-alpha	*tnf-α*	AJ413189	F	CAGGCGTCGTTCAGAGTCTC
			R	CTGTGGCTGAGAGCTGTGAG
Zeta-chain-associated protein kinase 70	*zap70*	MF175239	F	TGGTGAAGGAGGAGATGATGAGG
			R	GCGAACGATGTAGCGGTTGT

Table S2. Head kidney expression in response in gilthead seabream at time ø and fed dietary treatments for 2 weeks and 4 weeks. All values are reported as mean ± SE (n=9) (Raw data). P-values from two-way ANOVA (p ≤ 0.05). Tukey post-hoc test was used to identify differences in the experimental treatments. Different lowercase letters stand for significant differences between dietary treatments for the same time.

		**TØ**	**2 weeks**				**4 weeks**				**Two-way ANOVA (p<0.05)**		
Biological Process	Gene symbol	CTRL	CTRL	THR	TAU	HIS	CTRL	THR	TAU	HIS	Time	Diet	Time x diet
**Interleukins & Cytokines**	*il-1β*	0.08 ± 0.01	0.07 ± 0.01	0.10 ± 0.03	0.07 ± 0.01	0.05 ± 0.00	0.10 ± 0.03	0.07 ± 0.01	0.09 ± 0.02	0.10 ± 0.01	0.086	0.374	0.735
	il-6	0.04 ± 0.01	0.08 ± 0.02	0.06 ± 0.02	0.08 ± 0.01	0.04 ± 0.01	0.05 ± 0.01	0.05 ± 0.01	0.06 ± 0.01	0.05 ± 0.01	0.584	0.208	0.589
	il-7	1.06 ± 0.11	1.37 ± 0.16	1.14 ± 0.14	1.30 ± 0.21	1.07 ± 0.10	1.16 ± 0.09	1.14 ± 0.13	1.31 ± 0.14	1.08 ± 0.12	0.797	0.433	0.836
	il-8	0.07 ± 0.01	0.09 ± 0.01	0.08 ± 0.02	0.09 ± 0.01	0.08 ± 0.01	0.10 ± 0.02	0.08 ± 0.02	0.07 ± 0.01	0.08 ± 0.02	0.732	0.533	0.883
	il-10	0.69 ± 0.08	0.57 ± 0.04	0.68 ± 0.08	0.67 ± 0.07	0.61 ± 0.06	0.66 ± 0.03	0.62 ± 0.05	0.65 ± 0.06	0.69 ± 0.06	0.345	0.984	0.62
	il-12	0.09 ± 0.02	0.07 ± 0.01	0.05 ± 0.00	0.06 ± 0.01	0.06 ± 0.01	0.06 ± 0.01	0.05 ± 0.01	0.07 ± 0.02	0.06 ± 0.01	0.706	0.457	0.982
	il-15	0.25 ± 0.03	0.29 ± 0.04	0.26 ± 0.02	0.30 ± 0.03	0.28 ± 0.03	0.30 ± 0.02	0.28 ± 0.04	0.26 ± 0.03	0.27 ± 0.03	0.504	0.493	0.695
	il-34	1.57 ± 0.15	1.78 ± 0.12	1.76 ± 0.24	1.54 ± 0.10	1.37 ± 0.11	1.57 ± 0.13	1.39 ± 0.09	1.66 ± 0.07	1.66 ± 0.15	0.909	0.092	0.173
	tnf-α	0.25 ± 0.03	0.27 ± 0.03	0.24 ± 0.02	0.26 ± 0.02	0.24 ± 0.02	0.26 ± 0.01	0.22 ± 0.02	0.22 ± 0.01	0.23 ± 0.02	0.175	0.66	0.806
**Macrophages and monocytes chemokines**	*csf1r1*	4.30 ± 0.35	4.65 ± 0.39	4.40 ± 0.36	4.94 ± 0.48	4.48 ± 0.29	4.34 ± 0.32	4.09 ± 0.32	3.74 ± 0.14	4.59 ± 0.36	0.127	0.449	0.458
	ccr3	4.86 ± 0.55	5.94 ± 0.49	5.44 ± 0.40	5.17 ± 0.59	4.60 ± 0.40	5.71 ± 0.22	5.77 ± 0.25	5.83 ± 0.41	5.72 ± 0.49	0.094	0.038	0.548
	ck8/ccl20	0.76 ± 0.06	0.72 ± 0.08	0.81 ± 0.09	0.81 ± 0.06	0.74 ± 0.07	0.82 ± 0.07	0.78 ± 0.06	0.93 ± 0.11	0.84 ± 0.07	0.114	0.713	0.738
**Immunoglobulins**	*sIgM*	185.25 ± 27.90	188.57 ± 10.54	243.04 ± 28.52	225.26 ± 13.57	255.46 ± 23.91	223.21 ± 29.19	266.64 ± 25.54	249.75 ± 17.01	237.76 ± 13.26	0.224	0.23	0.817
	IgM-m	34.45 ± 3.82	25.76 ± 1.35	27.40 ± 1.86	32.16 ± 3.05	25.05 ± 1.39	31.24 ± 1.22	30.43 ± 2.00	29.85 ± 1.54	31.99 ± 2.74	0.03	0.579	0.283
	sIgT	0.28 ± 0.12	0.24 ± 0.15	0.07 ± 0.06	0.14 ± 0.09	0.24 ± 0.16	0.26 ± 0.13	0.82 ± 0.23	0.39 ± 0.16	0.17 ± 0.10	0.009	0.827	0.416
	IgT-m	6.67 ± 0.88	5.70 ± 0.81	6.62 ± 0.93	8.17 ± 1.76	8.21 ± 0.84	9.23 ± 1.74 **ab**	11.54 ± 1.92 **b**	5.16 ± 0.64 **a**	8.37 ± 0.79 **ab**	0.125	0.408	0.013
**Complement factor**	*c3*	0.02 ± 0.01	0.03 ± 0.02	0.00 ± 0.00	0.01 ± 0.00	0.02 ± 0.02	0.04 ± 0.03	0.04 ± 0.03	0.02 ± 0.01	0.02 ± 0.01	0.118	0.653	0.23
**Iron recycling**	hepc	15.00 ± 3.06	48.50 ± 9.73	49.69 ± 13.49	27.58 ± 4.61	43.92 ± 5.66	56.42 ± 9.67	53.29 ± 10.80	47.96 ± 11.24	45.01 ± 9.13	0.203	0.294	0.755
**T-cell markers**	*cd3x*	3.97 ± 1.22	2.43 ± 0.13	2.46 ± 0.12	2.55 ± 0.22	2.70 ± 0.23	2.41 ± 0.11	2.23 ± 0.14	2.31 ± 0.17	2.50 ± 0.28	0.302	0.714	0.955
	cd4-full	3.56 ± 1.41	1.97 ± 0.12	1.61 ± 0.05	1.77 ± 0.14	1.97 ± 0.15	1.94 ± 0.17	1.95 ± 0.14	1.86 ± 0.21	1.56 ± 0.20	0.762	0.476	0.29

	cd8a	4.28 ± 2.23	1.82 ± 0.25	1.69 ± 0.21	1.76 ± 0.17	1.73 ± 0.27	1.76 ± 0.17	1.78 ± 0.15	1.93 ± 0.17	1.96 ± 0.34	0.653	0.927	0.987
	cd8b	1.34 ± 0.81	0.47 ± 0.07	0.41 ± 0.05	0.42 ± 0.04	0.48 ± 0.10	0.44 ± 0.05	0.48 ± 0.07	0.57 ± 0.10	0.41 ± 0.08	0.776	0.58	0.697
	zap70	2.38 ± 0.44	1.83 ± 0.17	1.87 ± 0.10	1.95 ± 0.18	2.30 ± 0.30	2.05 ± 0.15	1.91 ± 0.17	1.67 ± 0.15	1.89 ± 0.21	0.472	0.427	0.422
**Pattern recognition receptors**	*tlr2*	3.93 ± 0.33	3.55 ± 0.31	3.10 ± 0.19	3.52 ± 0.37	3.13 ± 0.19	3.37 ± 0.25	3.20 ± 0.18	2.94 ± 0.27	3.64 ± 0.27	0.845	0.449	0.282
	tlr5	0.35 ± 0.05	0.33 ± 0.02	0.36 ± 0.02	0.31 ± 0.01	0.34 ± 0.02	0.32 ± 0.02	0.37 ± 0.02	0.35 ± 0.01	0.38 ± 0.03	0.409	0.041	0.622
	tlr9	1.70 ± 0.21	0.86 ± 0.12	0.97 ± 0.07	1.05 ± 0.14	0.92 ± 0.10	0.97 ± 0.08	0.81 ± 0.05	1.00 ± 0.13	1.11 ± 0.09	0.676	0.696	0.22
	mrc1	6.34 ± 0.75	7.62 ± 0.22	7.75 ± 0.57	7.27 ± 0.53	7.38 ± 0.82	8.08 ± 0.43	7.33 ± 0.43	8.22 ± 0.51	8.32 ± 0.44	0.212	0.791	0.57
**Caspase**	*casp3*	1.00 ± 0.06	1.02 ± 0.08	1.09 ± 0.07	1.11 ± 0.11	1.15 ± 0.08	1.04 ± 0.11	1.08 ± 0.09	0.99 ± 0.06	0.93 ± 0.09	0.252	0.709	0.762
**Lectins**	*clec10a*	0.54 ± 0.11	1.22 ± 0.14 ^{b}	0.43 ± 0.05 ^{a}	1.18 ± 0.09 ^{ab}	0.83 ± 0.19 ^{ab}	1.22 ± 0.25	1.33 ± 0.19	0.84 ± 0.17	0.83 ± 0.17	0.258	0.066	<0.001
